# The Use of Unidirectional Barbed Suture for Urethrovesical Anastomosis during Robot-Assisted Radical Prostatectomy: A Systematic Review and Meta-Analysis of Efficacy and Safety

**DOI:** 10.1371/journal.pone.0131167

**Published:** 2015-07-02

**Authors:** Huixin Li, Chunxiao Liu, Haibin Zhang, Wenfeng Xu, Jianhua Liu, Yong Chen, Tangxuan Li, Bin Li, Zhenquan Wu, Taolin Xia

**Affiliations:** 1 Department of Urology, the First People's Hospital of Foshan (Foshan Affiliated Hospital of Sun Yat-sen University), Foshan, Guangdong, People's Republic of China; 2 Department of Urology, Zhujiang Hospital, Southern Medical University, Guangzhou, Guangdong, People's Republic of China; Eberhard Karls University, GERMANY

## Abstract

**Background:**

Unidirectional barbed suture (UBS) has been widely used for surgery in recent years, especially for urethrovesical anastomosis (UVA) during robot-assisted radical prostatectomy (RARP). However, the efficacy and safety comparing it with conventional non-barbed suture (CS) for UVA is still controversial.

**Aims:**

The objective of this study is to assess the current evidence regarding the efficacy and safety of UBS compared with CS for UVA during RARP.

**Methods:**

We comprehensively searched PubMed, Embase, The Cochrane Library, SinoMed (Chinese) and other databases on Oct. 9, 2014 to conduct a systematic review and meta-analysis of all randomized controlled trials (RCTs) and other comparative studies evaluating these two types of suture. The outcome measures included anastomosis time operative time, posterior reconstruction (PR) time, postoperative leakage (PL) rate and continence rates at different time points (4-6 weeks, 3 months, 6-12 months) after surgery. Secondary outcomes included estimated blood loss (EBL) and length of catheterization (LOC).

**Results:**

Three RCTs and six observational studies including 786 cases were identified. Meta-analysis of extractable data showed that use of UBS could significantly reduce anastomosis time (weighted mean difference [WMD]:-3.98min; 95% confidence interval [CI], -6.02 -1.95; p = 0.0001), operative time (WMD:-10.06min; 95% CI, -15.45–-4.67; p = 0.0003) and PR time (WMD:-0.93min; 95% CI, -1.52–-0.34; p = 0.002). No significant difference was found in PL rate, EBL, LOC, or continence rates at 4-6 weeks, 3 months and 6–12 months after surgery.

**Conclusions:**

Our meta-analysis indicates that UBS appears to be safe and efficient as CS for UVA during RARP with not only shorter anastomosis time, operative time, PR time, but also equivalent PL rate, EBL, LOC, and continence rates at 4-6 weeks, 3 months and 6-12 months after surgery. For the inherent limitations of the eligible studies, future more persuasive RCTs are needed to confirm and update our findings.

## Introduction

Nowadays, robot-assisted radical prostatectomy (RARP) has become one of the primary procedures for performing radical prostatectomy in western countries since its first use in the United States in 2001 [1,2], while urethrovesical anastomosis (UVA) is still the most critical and challenging step of it [3]. And creation of a watertight, optimally apposed, tension-free, and well supported VUA remains the cornerstone for postoperative recovery [4]. The most common suture materials for UVA at present are Monocryl (poliglecaprone-25, J and J Medical, Somerville, NJ, USA) monofilament suture and Vicryl (Polyglactin-910; Ethicon, J and J Medical, Somerville, NJ, USA) braided suture, especially the former. Though the widely used Van Velthoven stitch has been adopted to make UVA easier [5], it has limitations relating to slippage of the conventional suture, necessitating constant traction by an assistant or repeated tightening by the surgeon. Recently, a poly-glyconate, unidirectional barbed synthetic absorbable suture (V-Loc Wound Closure Device, Covidien, Mansfield, MA) has been re-developed and made available commercially, and the V-Loc 180 absorbable wound closure device is mostly used within it for UVA.

Prepared by copolymer of glycolic acid and trimethylene carbonate, the device consists of a unidirectional barbed absorbable thread, armed with a surgical needle at one end and a welded-loop end effector at the other. The barbs are evenly-spaced throughout the strand, providing secure closure of the incision by distributing tension across the anastomotic stoma. Compared to the conventional non-barbed synthetic absorbable suture, this design prevents slippage, precludes the need for assistance or knot tying and avoids tissue tearing and ischemia. It has tensile strength of 80% after 7 days, 75% after 14 days, 65% after 21 days, and is fully absorbed by 180 days.

Since its first application for UVA in 2009, UBS has been widely used for UVA gradually. Several studies comparing it and conventional non-barbed suture (CS) have been reported. However, most are small series and some have conflicting results. Therefore it's necessary for us to search the available literature systemically and evaluate the effectiveness and safety of UBS versus CS for UVA during RARP.

## Materials and Methods

### Search Strategy and Study Selection

A systematic review and meta-analysis was designed and conducted according to the Preferred Reporting Items for Systematic Reviews and Meta-analysis (PRISMA) and Meta-analysis of Observational Studies in Epidemiology recommendations (MOOSE) for study reporting [6,7].

A literature search was performed on Oct. 9, 2014 without restriction to regions, languages, or publication types. The primary sources were the electronic databases of PubMed, Embase, The Cochrane Library and SinoMed(Chinese). The following MeSH terms were searched with their combinations in [Full text]: *barbed / V-Loc / self-retaining, anastomosis / VUA*, *reconstructive / reconstruction, *and *radical prostatectomy*. Trials registered in the ClinicalTrials.gov, Australian or Netherlands clinical trials registry, and World Health Organization database, CKNI (Chinese) and VIP (Chinese) were also searched. The related articles function was also used to broaden the search, and the computer search was replenished with manual searches of the reference lists of all retrieved studies. If multiple reports were available from the same population, the most recent or complete one was selected.

All available randomized controlled trials (RCTs) and observation studies (prospective cohort, retrospective cohort or case-control studies) that compared UBS with non-barbed suture for UVA during RARP, which had at least one of our primary and secondary outcomes, were included. Editorials, letters to the editor, case reports, review articles, conference abstracts, and animal experimental studies were excluded.

### Data Extraction

Data from the included studies were extracted carefully and independently by two of the authors (HZ and WX). Any disagreement was resolved by the adjudicating senior author (CL).

The primary outcomes were anastomosis time, operative time, posterior reconstruction (PR) time, postoperative leakage (PL) rate and continence rates at different time points (4–6 weeks, 3 months, 6–12 months) after surgery. Secondary outcomes included estimated blood loss (EBL) and length of catheterization (LOC). Besides PL and incontinence, Meta-analysis was not performed on other UVA related complication rates and suture cost for insufficient data from the researches.

### Assessment of Study Quality

Studies were rated for the level of evidence according to criteria provided by the Centre for Evidence-Based Medicine in Oxford, UK [8]. The methodological quality of RCTs was assessed by the Cochrane risk of bias tool [9], while that of other studies was classified by the modified Newcastle-Ottawa scale (NOS) [10,11], which consists of three factors: patient selection, comparability of the study groups, and assessment of outcome. A score of 0–9 (allocated as stars) was assigned to each study except RCTs (Table 1).

**Table 1:** Characteristics of included studies.

Study	Level of evidence	Design	Patients, no. UBS CS	Suture technique	Materials of CS	PR	Match factors*	Follow up^#^, mo.	Quality score
Desai et al[14]	3b	P	113 76	VV	3–0 Monocryl	Y	1,2,3,7,10,11	3	★★★★★
Hemal et al[15]	3b	RP	25 25	VV	3–0 Monocryl	N	1,2,5,6,7,9,10	6	★★★★★★
Manganiello et al[16]	3b	P	35 35	VV	3–0 Monocryl	N	1,2,3,5,6,7,10,11	5	★★★★★★
Massoud et al[17]	3b	P	40 40	BS with VV CS with IS	3–0 Vicryl	N	1,4,5,9	12	★★★★★
Polland et al[18]	3b	RP	42 42	VV	3–0 Monocryl	Y	1,2,7,9	9.4 (7–13)	★★★★★★★
Sammon et al[19]	2b	RCT	33 31	VV	3–0 Monocryl	Y	1,2,3,4,6,7,8,10	9.1	RCT
Tewari et al[20]	3b	P	50 50	VV	3–0 Monocryl	Y	1,2,3,5,6,7,9,10,11	NA	★★★★★★★
Williams et al[21]	2b	RCT	45 36	VV	3–0 Vicryl	N	1,2,3,6,7,8,9,10,11	NA	RCT
Zorn et al[22]	2b	RCT	34 34	VV	3–0 Monocryl	Y	1,2,3.4,5,6,7,8,9,10,11	6.2	RCT

UBS = unidirectional barbed suture; CS = conventional non-barbed suture; PR = posterior reconstruction; VV = Van Velthoven technique; IS = interrupted suture; P = prospective; RP = retrospective design, prospective data collection; RCT = randomized control trial; Y = yes; N = no; NA = data not available. 3–0 Monocryl = 3–0 Monocryl (poliglecaprone-25, J and J Medical, Somerville, NJ, USA) monofilament suture. 3–0 Vicryl = 3–0 Vicryl (Polyglactin-910; Ethicon, J and J Medical, Somerville, NJ,USA) braided suture.

^**#**^Mean and median.

*Matching: 1 = age; 2 = PSA; 3 = body mass index; 4 = AUA-SS/IPSS, American Urological Association Symptom Score; 5 = prostate volume; 6 = Biopsy/Pathology Gleason score; 7 = clinical/Pathological stage; 8 = Medical history; 9 = single surgeon; 10 = estimated blood loss; 11 = positive margin rates.

### Statistical Analysis

All the data were analyzed using Review Manager 5.2.7 (Cochrane Collaboration, Oxford, UK). Quantitative synthesis was done when more than one eligible study was identified. Where appropriate, a combined estimate of treatment effect across similar studies was calculated for each prespecified outcome. The weighted mean difference (WMD) and odds ratio (OR) were used to compare continuous and dichotomous variables, respectively. All results were reported with 95% confidence intervals (CIs). For studies that presented continuous data as medians and range values, the WMD and standard deviations were calculated using the technique described by Hozo et al [12].

Methodological heterogeneity was assessed during selection, while statistical heterogeneity between studies was assessed using the chi-square test with significance set at p < 0.10 and quantified by I^2^ statistic. The random-effects model was used if there was high heterogeneity (I^2^≥50%); otherwise, the fixed-effects model was used [9].

Subgroup analysis was performed to subdivide the included studies according to the procedure with or without PR, which was defined as using running or interrupted suture to re-approximating posterior rhadbosphincter and Denonvilliers' fascia before UVA. Sensitivity analysis was performed for studies excluding the influence of the learning curve and different suture methods.

## Results

### Literature Search

**Fig 1:**
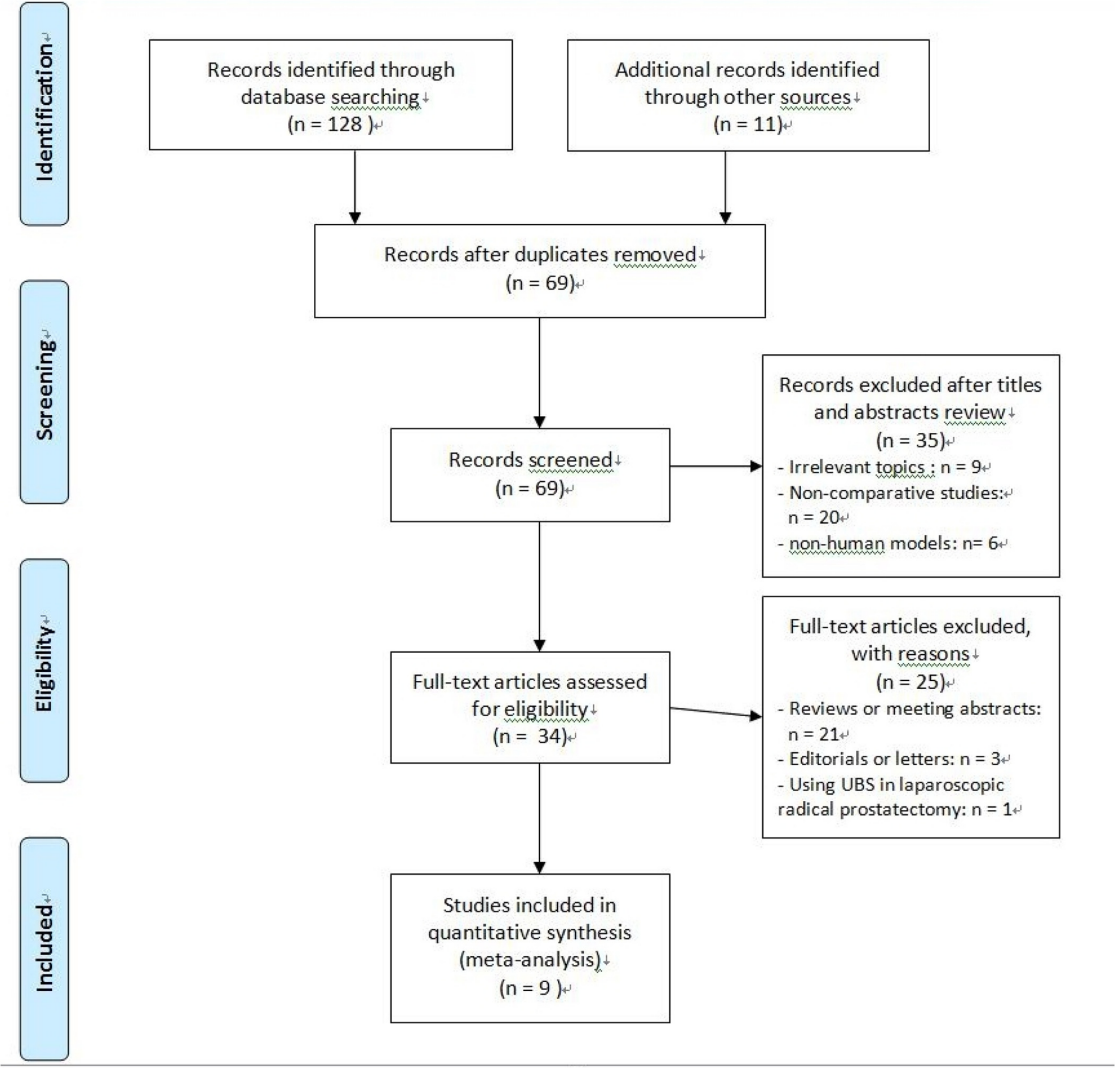
PRISMA flow diagram of studies identified, included, and excluded.

Fig 1 shows the flow diagram of studies identified, included and excluded. 139 studies were selected using the predefined search strategy. Among them, 70 studies were excluded for duplication. Then after removal of another 35 studies by careful reading the titles and abstracts, full text of the remaining 34 studies was evaluated in detail. Three editorials, 21 reviews or conference abstracts were excluded. Besides, one study [13] comparing the two sutures during endoscopic extraperitoneal radical prostatectomy was not included. Finally, nine studies [14–22] including 786 patients (417 cases for using UBS and 369 cases for using CS) were included in the final meta-analysis. All of these publications were full-text articles. Examination of the references listed in them did not yield any further studies for evaluation. Agreement between the two reviewers was 96% for study selection and 93% for quality assessment.

### Characteristics of the Included Studies

Table 1 shows the characteristics of included studies. Among them, there were three RCTs (LE: 2b) [19,21,22], four prospective studies compared contemporary series of patients (LE: 3b) [14,16,17,20] and two retrospective studies declared prospective data collection (LE: 3b) [15,18]. Considering different CS materials, seven studies used Monocryl suture in the control group [14–16,18–20,22], while two studies used Vicryl suture [17,21], respectively. In addition, five studies performed PR [14,18–20,22], while the remaining studies didn't [15–17, 21]. All the studies used da Vinci Surgical System to perform RARP.

The quality of included studies was generally moderate. True randomization was used in three RCTs [19,21,22], while another four comparative studies[15,16,18,20] achieving six or more stars according to the modified NOS were considered to be of high quality. Most of the studies adopted appropriate protocols for treatment assignment with rigorous and normative allocation. Match factors in all researches were comparable. However, only three RCTs considered the surgeon's experience in using UBS, and just seven studies described the length of follow-up [14–19, 22]. Except for postoperative leakage and incontinence, the two most crucial UVA related complications, most of the studies provided insufficient data of other perioperative, postoperative complications and suture cost. Intention-to-treat analyses and methods for dealing with missing data were adequately described in the majority of studies. In addition, selective reporting was not found during our analysis.

### Primary Outcomes

**Fig 2:**
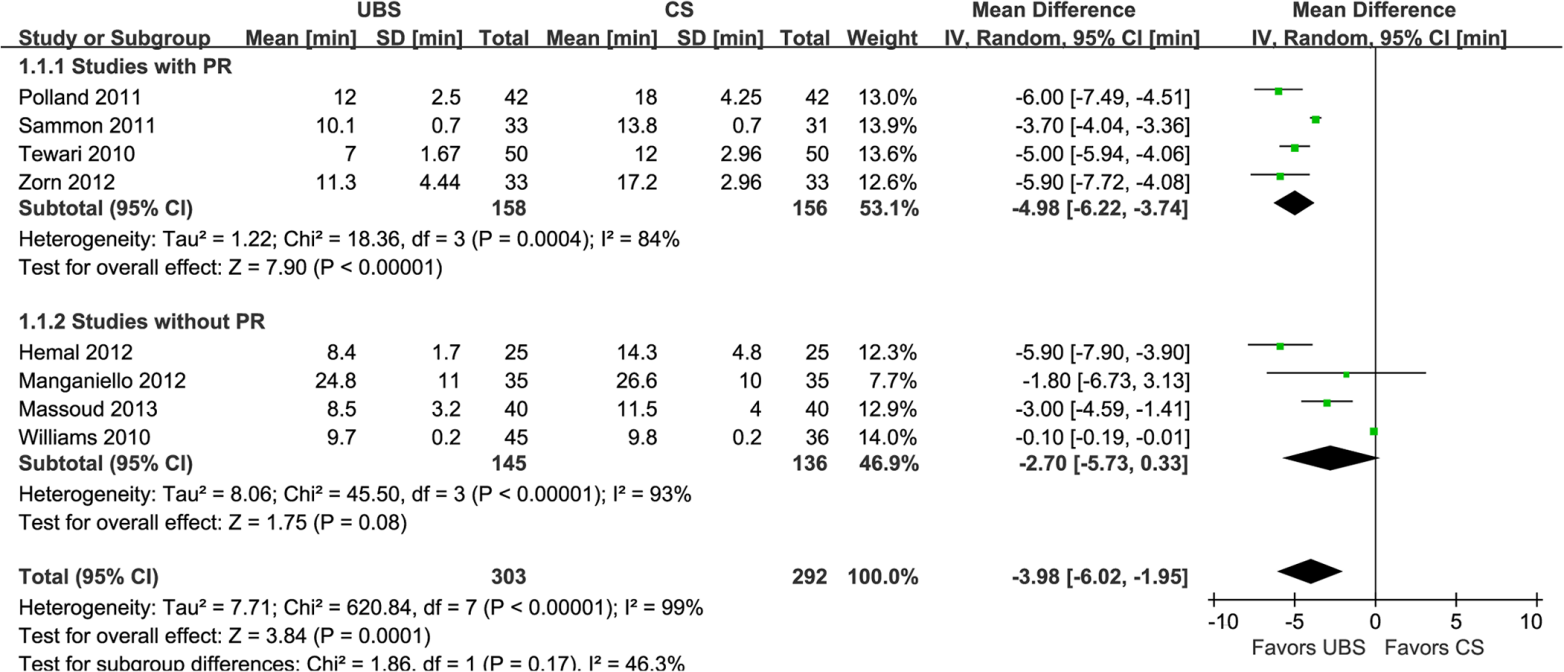
Forest plot and meta-analysis of anastomosis time. UBS = unidirectional barbed suture; CS = conventional non-barbed suture; SD = standard deviation; IV = inverse variance method; CI = confidence interval.

**Fig 3:**
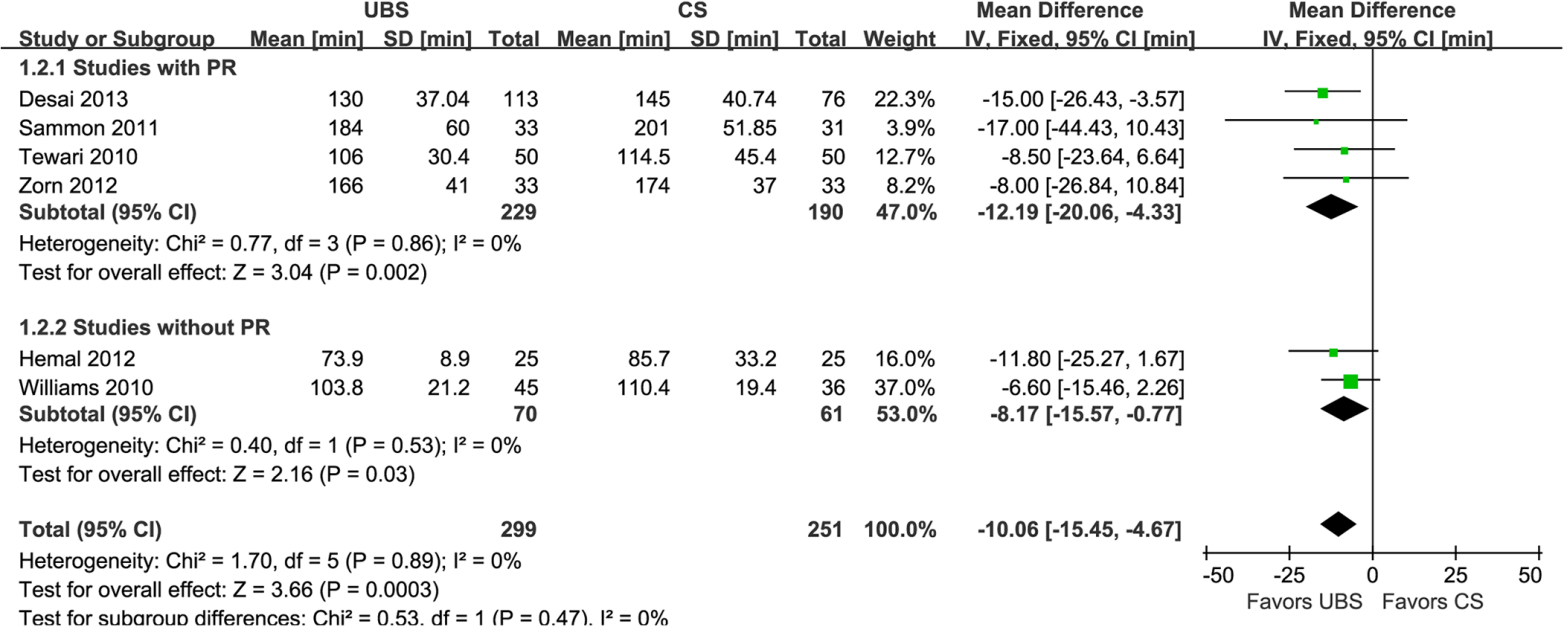
Forest plot and meta-analysis of operative time. UBS = unidirectional barbed suture; CS = conventional non-barbed suture; SD = standard deviation; IV = inverse variance method; CI = confidence interval.

**Fig 4:**
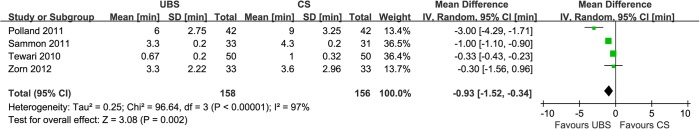
Forest plot and meta-analysis of posterior reconstruction time. UBS = unidirectional barbed suture; CS = conventional non-barbed suture; SD = standard deviation; IV = inverse variance method; CI = confidence interval.

Results of combined estimates comparing UBS versus CS are shown in Table 2. Eight studies demonstrated the anastomosis time was significantly shorter in UBS group than CS group (WMD:-3.98min; 95% CI, -6.02–-1.95; p = 0.0001) (Fig 2) [15–22]. Of six studies reported operative time [14,15,19–22], the results showed that it was deceased with UBS than CS (WMD:-10.06min; 95% CI, -15.45–-4.67; p = 0.0003) (Fig 3). Five studies performed PR using Rocco stitch [23, 24], but just four of them reported the RP time for meta-analysis [18–20, 22]. And it was significantly reduced with UBS, either (WMD:-0.93min; 95% CI, -1.52–-0.34; p = 0.002) (Fig 4).

**Table 2:** Results of meta-analysis comparing UBS and CS.

Outcomes of interest	Results of the combined studies					Study heterogeneity			
	Studies no.	UBS patients no.	CS patients no.	WMD/OR^#^ (95%CI)	*p* value*	X^2^	df	I^2^	*p* value*
**Primary outcomes**									
Anastomosis time,min	8	303	292	-3.98 (-6.02–-1.95)	**0.0001**	620.84	7	99%	**<0.00001**
Operative time,min	6	299	251	-10.06(-15.45–-4.67)	**0.0003**	1.70	5	0%	0.89
Posterior reconstruction time,min	4	158	156	-0.93(-1.52–0.34)	**0.002**	96.64	3	97%	**<0.00001**
Postoperative leakage rate	4	226	178	2.17^**#**^ (0.86–5.50)	0.10	3.5	3	14%	0.32
Continence rate at 4–6 weeks after surgery	3	106	103	1.19^**#**^ (0.69–2.08)	0.53	0.08	2	0%	0.96
Continence rate at 3 months after surgery	2	146	109	0.93^**#**^ (0.55–1.59)	0.80	0.65	1	0%	0.42
Continence rate at 6–12 months after surgery	3	107	108	1.56^**#**^ (0.60–4.04)	0.36	0.06	2	0%	0.97
**Secondary outcomes**									
Estimated blood loss,ml	7	334	286	9.33(-7.56–26.21)	0.28	13.32	6	55%	**0.04**
Length of catheterization,d	5	188	177	-0.14(-0.80–0.51)	0.67	31.05	4	87%	**<0.00001**

UBS = unidirectional barbed suture; CS = conventional non-barbed suture; WMD/OR = weighted mean difference/odds ratio;df = degrees of freedom; CI = confidence interval

^*^Statistically significant results are showed in bold

^#^odds ratio.

**Fig 5:**
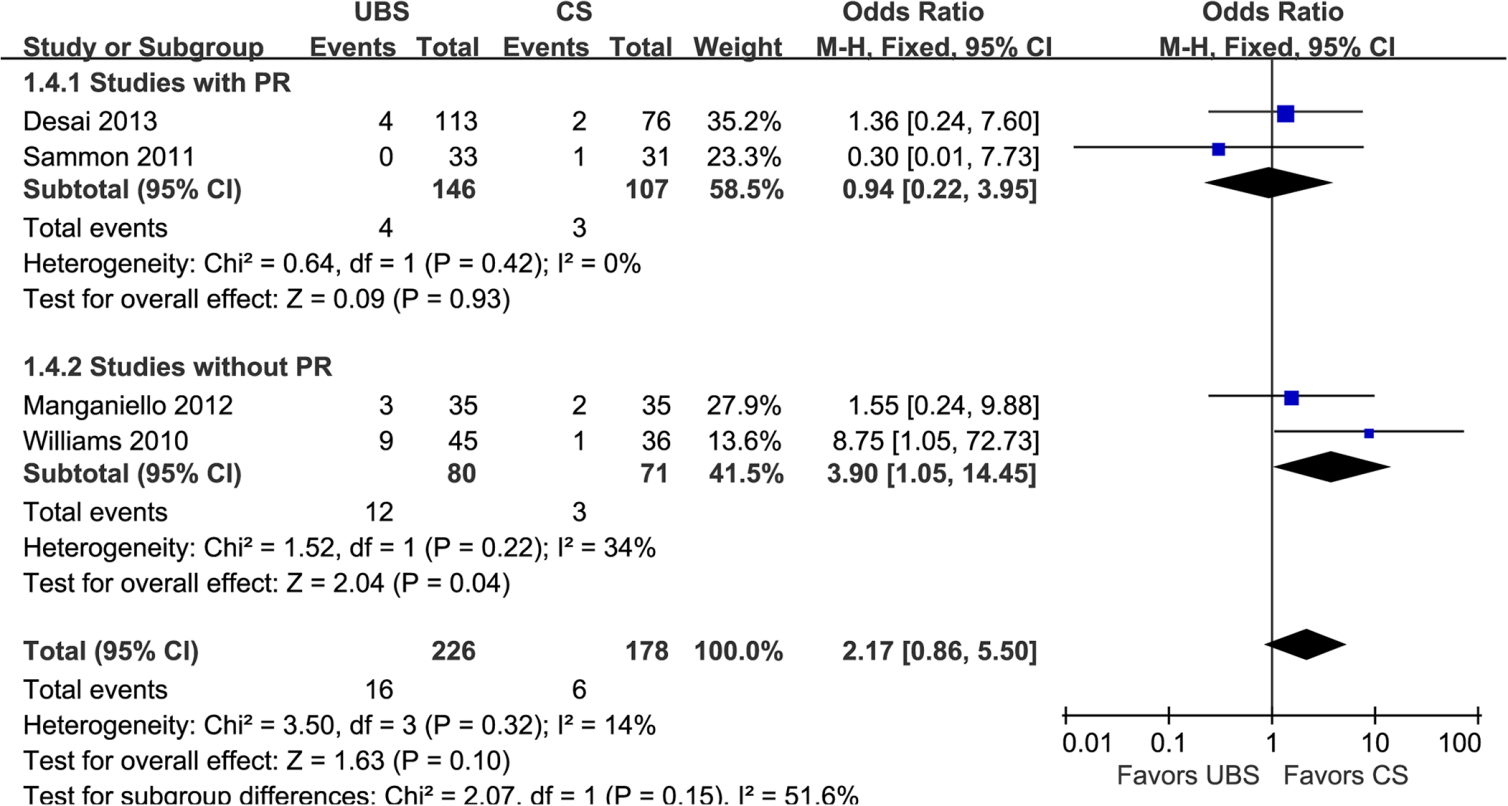
Forest plot and meta-analysis of postoperative leakage rate. UBS = unidirectional barbed suture; CS = conventional non-barbed suture; SD = standard deviation; M-H = Mantel-Haenszel method; CI = confidence interval.

PL was defined as contrast extravasation presented in cystogram 5–12 days postoperatively. Four studies used cystogram to evaluate PL and showed it was no significant difference between the two groups (7.08% and 3.37%; OR: 2.17; 95% CI, 0.86–5.50; p = 0.10) (Fig 5) [14,16, 19,21].

**Fig 6:**
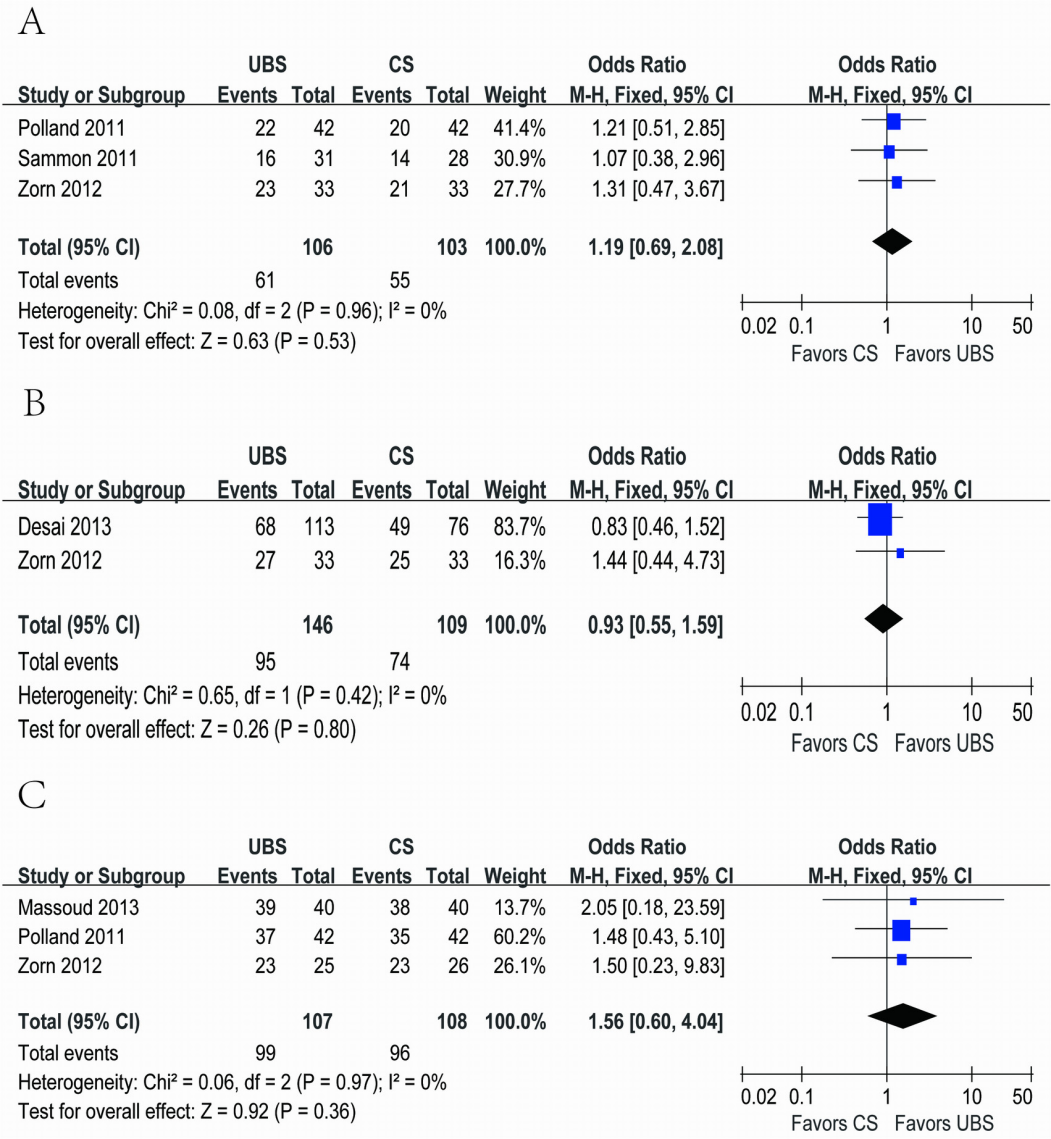
Forest plot and meta-analysis of continence rate at (A) 4–6 weeks, (B) 3 months and (C) 6–12 months after surgery. UBS = unidirectional barbed suture; CS = conventional non-barbed suture; SD = standard deviation; M-H = Mantel-Haenszel method; CI = confidence interval.

Continence was defined as patients had no need for pads usage per day postoperatively. Three [18,19,22], two [14, 22] and three [17,18,22] studies reported continence rate using the same judging criteria at 4–6 weeks, 3 months and 6–12 months after surgery, respectively. And none of them showed significant difference between the two groups (57.5% and 53.4%; OR: 1.19; 95% CI, 0.69–2.08; p = 0.53; 65.1% and 67.9%; OR: 0.93; 95% CI, 0.55–1.59; p = 0.80; 92.5% and 88.9%; OR: 1.56; 95% CI, 0.60–4.04; p = 0.36, respectively) (Fig 6A, 6B and 6C).

### Secondary Outcomes

**Fig 7:**
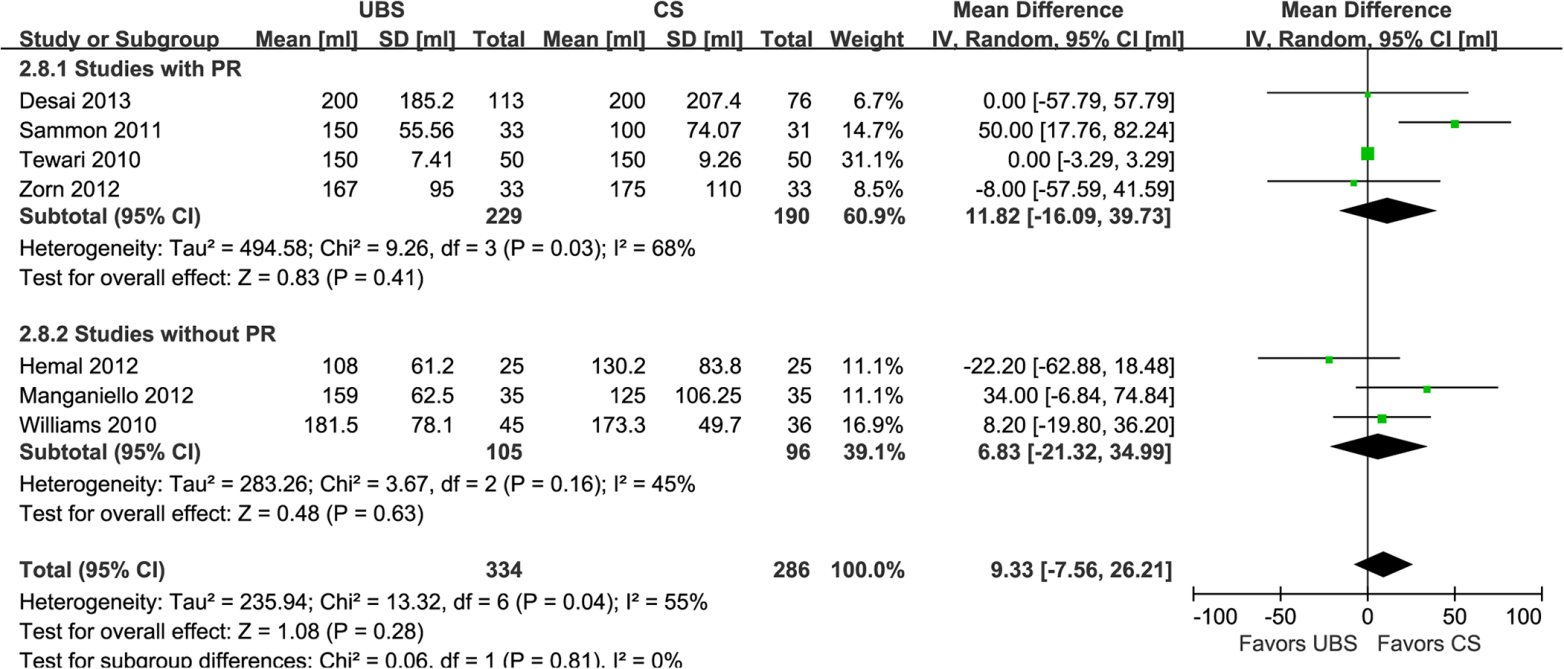
Forest plot and meta-analysis of estimated blood loss. UBS = unidirectional barbed suture; CS = conventional non-barbed suture; SD = standard deviation; IV = inverse variance method; CI = confidence interval.

**Fig 8:**
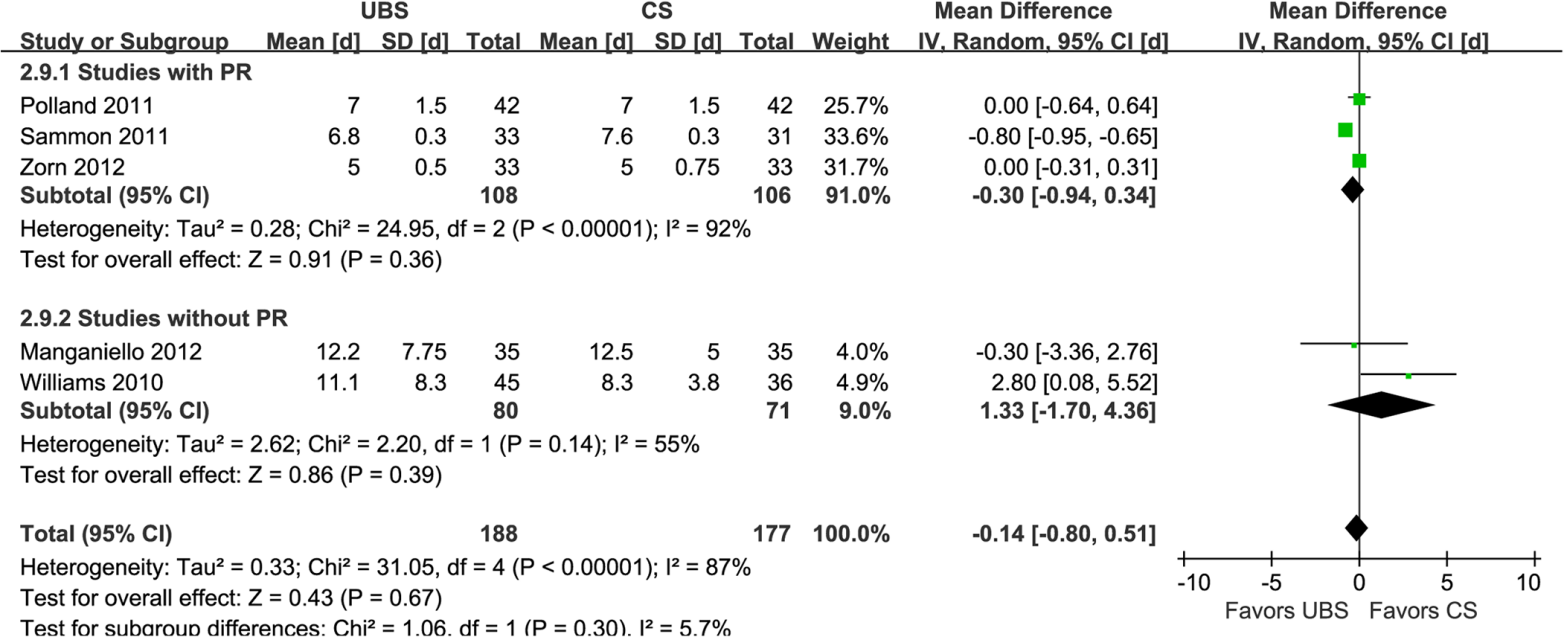
Forest plot and meta-analysis of length of catheterization. UBS = unidirectional barbed suture; CS = conventional non-barbed suture; SD = standard deviation; IV = inverse variance method; CI = confidence interval.

EBL is regarded as one of the most important security indices for a procedure. Seven studies showed that there was no significant difference of it between UBS and control group (WMD:9.33ml; 95% CI, -7.56–26.21; p = 0.28) (Fig 7) [14–16,19–22]. Reported in five studies [16,18,19,21,22], LOC was not found to be significant difference between the two groups, either (WMD:-0.14d; 95% CI, -0.80–0.51; p = 0.67) (Fig 8).

### Subgroup Analysis

Concerning the results above-mentioned showed using UBS could significantly reduce PR time, which might influence the other outcomes, we divided the included studies into subgroups on the basis of performing PR or not. However, in that a small amount of studies were included to evaluate continence rate in each time period, subgroup analysis was not performed to this outcome.

In the subgroup analysis of studies with RP [14,18–20,22], there were similar to the original analysis in anastomosis time (WMD: -4.98min; 95% CI, -6.22–3.74; p<0.00001)(Fig 2), operative time (WMD: -12.19min; 95% CI, -20.06–-4.33; p = 0.002)(Fig 3), PL rate (2.74% and 2.80%; OR: 0.94; 95% CI, 0.22–3.95; p = 0.93)(Fig 5), EBL (WMD:11.82ml; 95% CI, -16.09–39.73; p = 0.41)(Fig 7), and LOC (WMD:-0.30d; 95% CI, -0.94–0.34; p = 0.36)(Fig 8). In the other subgroup of studies without RP [15–17,21], there were differences compared with the original analysis in anastomosis time (WMD:-2.73min; 95% CI, -5.73–0.33; p = 0.08)(Fig 2) and PL rate (15.0% and 4.23%; OR: 3.90; 95% CI, 1.05–14.45; p = 0.04)(Fig 5), whereas it showed no difference in operative time (WMD: -8.17min; 95% CI, -15.57–-0.77; p = 0.03)(Fig 3), EBL (WMD:6.83ml; 95% CI, -21.32–34.99; p = 0.63)(Fig 7) or LOC (WMD:1.33d; 95% CI, -1.70–4.36; p = 0.39)(Fig 8).

### Heterogeneity and Sensitivity Analysis

There was a low estimate of statistical heterogeneity in operative time (I^2^ = 0%), PL rate (I^2^ = 14%), and all of the continence rates (I^2^ = 0%), while high heterogeneity existed in anastomosis time (I^2^ = 99%), PR time (I^2^ = 97%), LOC (I^2^ = 87%) and EBL (I^2^ = 55%), respectively. Considering learning curve might be one of most critical impact factors, we excluded one RCT [21] in which the surgeon had probably little experience when using UBS in the first 29 patients (he then modified his technique for the subsequent patients). Besides, applying different suture methods might be another important impact factor to our results. So we excluded another study [16] that using interrupted suture in CS group. Coincidently, only these two studies used Polyglactin-910 braided suture for UVA in the control group among the included studies. After excluding them for sensitivity analysis, heterogeneity decreased in anastomosis time (I^2^ = 78%) and PL rate (I^2^ = 0%), and the results were equivalent to the original analysis (Table 3). However, it didn't decrease in PR time, which was not performed in these two studies. In addition, the heterogeneity of EBL and LOC after sensitivity analysis went near to that before, suggesting that these two factors might not have much impact on them. Publication bias was kept to a minimum according to our search strategy, and funnel plot was not displayed because the total number of included studies was less than 10.

**Table 3:** Sensitivity analysis comparing UBS and CS.

Outcomes of interest	Results of the combined studies					Study heterogeneity			
	Studies no.	UBS patients no.	CS patients no.	WMD/OR^#^ (95%CI)	*p* value*	X^2^	df	I^2^	*p* value*
**Primary outcomes**									
Anastomosis time,min	6	218	216	-4.97 (-6.09–3.86)	**<0.00001**	22.54	5	78%	**0.0004**
Operative time,min	5	254	215	-12.09(-18.88–-5.30)	**0.0005**	0.77	4	0%	0.94
Posterior reconstruction time,min	4	158	156	-0.93(-1.52–0.34)	**0.002**	96.64	3	97%	**<0.00001**
Postoperative leakage rate	3	181	142	1.14^**#**^ (0.37–3.52)	0.83	0.79	2	0%	0.67
Continence rate at 4–6 weeks after surgery	3	106	103	1.19^**#**^ (0.69–2.08)	0.53	0.08	2	0%	0.96
Continence rate at 3 months after surgery	2	146	109	0.93^**#**^ (0.55–1.59)	0.80	0.65	1	0%	0.42
Continence rate at 6–12 months after surgery	2	67	68	1.49^**#**^ (0.53–4.18)	0.45	0.00	1	0%	0.99
**Secondary outcomes**									
Estimated blood loss,ml	6	289	250	9.93(-11.31–31.17)	0.36	13.04	5	62%	**0.02**
Length of catheterization,d	4	143	141	-0.30(-0.92–0.32)	0.34	24.99	3	88%	**<0.0001**

UBS = unidirectional barbed suture; CS = conventional non-barbed suture; WMD/OR = weighted mean difference/odds ratio; df = degrees of freedom; CI = confidence interval

^*^Statistically significant results are showed in bold

^#^odds ratio.

## Discussion

Our systematic review and meta-analysis of three RCTs and six observational studies including 786 patients showed that UBS was safe with significantly reduced anastomosis time, operative time and PR time compared to CS for UVA during RARP. No significant difference was found in PL rate, EBL, LOC, or continence rates at 4–6 weeks, 3 months and 6–12 months after surgery.

As we all know, safety and efficacy are always put in the first place in the application of any new suture material. The pooled data indicates that UBS is as safe and effective as CS for UVA in RARP. Our analysis demonstrated that the anastomosis time was significantly reduced in UBS group than CS group (WMD:-3.98min; 95% CI, -6.02–-1.95; p = 0.0001). However, when subgroup analysis was performed, the significant difference was just found in the subgroup with PR. We speculate that the approximation of Denonvilliers' fascia and the posterior urethral rhabdosphincter brings the bladder neck and urethral stump into close proximity, facilitating the completion of delicate tension-free anastomosis [25]. Nevertheless, there was still a trend of statistical significance that favoured using UBS in the subgroup without PR. Although the heterogeneity of anastomosis time among studies was quite high, it did decrease when we made a sensitivity analysis to exclude the influence of surgeon experience and different suture methods. And the anastomosis time in all the subgroup turned out to be significantly shorter with UBS in sensitivity analysis. Nevertheless, according to our data from 8 studies, the average time for UVA with UBS was 11.27min, while that with CS was 15.25min. Therefore, the absolute time difference for anastomosis was 3.98min (a 26.1% reduction in the anastomosis time), which is of clinical significance.

PR time was also significantly deceased in UBS group than control group (WMD:-0.93min; 95% CI, -1.52–-0.34; p = 0.002). However, the heterogeneity among studies was high. By inference, it might be due to the different suture methods, number of stitches, surgeon’s experience, outcome measurement, and so on. Therefore, one should be cautious when using this result to make decisions in practice.

Interestingly, we found that the total operative time was significantly shorter in UBS group (WMD:-10.06min; 95% CI, -15.45–-4.67; p = 0.0003), no matter in subgroup or sensitivity analysis, though there was no significant difference in the majority of the included studies for this variable except one [14]. The explanation could be the difference did exist but we couldn't find it due to the relative small sample size in most researches. But the performance of a meta-analysis allows us to overcome this limitation by increasing statistical power to find the difference. The absolute time difference of 10.06min means a 7.25% reduction in total procedure time with UBS. Though not a long time, it’s valid for actually saving the charges for operation and good for patients to recover.

PL is one of the most important UVA relative complications of RARP, because it prolongs catheterization time and may cause peritonitis and ileus requiring bowel rest and parenteral nutrition as well as require image-guided drain placement [26]. The pooled analysis for PL rate showed that it was comparable between the two groups (7.08% and 3.37%; OR: 2.17; 95% CI, 0.86–5.50; p = 0.10), which indicated that using UBS was as safe as CS when concerned with PL. There seemed to be a trend that favored CS and the PL rate with UBS was inferior to CS in the subgroup without RP, but we can explain that it was due to the inexperience of the surgeon in one RCT [21]. We didn‘t find any difference when we excluded this to perform a sensitivity analysis (3.87% and 3.52%; OR: 1.14; 95% CI, 0.37–3.52; p = 0.83).

With regard to continence rate, the most critical functional outcome after RARP, we found equivalence between both groups at any time point above-mentioned. Considering that incontinence is also an important complication of RARP and will influence patients’ life quality seriously, surgeons always try their best to reduce it. Our result means that it is as secure and effective with UBS for UVA as CS in urinary continence after procedure. However, it still needs to be proven by more applications and long-term follow-up in future.

The pooled data of EBL and LOC using random effects showed that there was no significant difference between study group and control group, either. It illustrates that using UBS has no negative impact on blood loss and postoperative recovery.

However, our meta-analysis has some limitations that must be taken into account. First, the total number of studies and the quantity of RCTs in this field were relatively small, while the quality of included studies was just moderate. However, we undertook multiple strategies to identify studies, used rigorous criteria to match for the characteristics, applied strict criteria to evaluate the methodological quality of the studies, and perform subgroup and sensitivity analysis to minimize the heterogeneity and reduce confounding. Second, the data of some of the UVA related complications, like intraoperative leakage, urethral tear, urinary retention, bladder neck contracture, stone formation, and erosion of suture, were insufficient. Therefore, it was hard to calculate the complication rates in all treatment groups. Nevertheless, in our study we’ve compared PL rate and incontinence rate, two most important UVA related complications, and the results are favourable in both groups. Further researches are needed to capture those results. Third, due to the insufficient data, different suture technique and different fee scale in different area, it's hard to tell whether the total charge for UBS group was expensive or not. Although the cost of UBS material is generally higher compared with CS materials, the utilizing of UBS for UVA could significantly shorten the total operative time according to our analysis, which could lead to saving the charges for operating room occupancy and anesthesia. Fourth, the heterogeneity of some of the effect estimates was high, and more homogeneous RCTs with high quality are awaited to make a more comprehensive evaluation of UBS. Finally, the follow-up period was not long enough, so long-term outcomes of UBS, especially for long-term complication rates and functional outcomes, remain to be proven.

Nevertheless, to our knowledge, this study is the first systematic review and meta-analysis on the use of UBS for UVA during RARP. Given that it has been used in urologic surgery for more than 4 years and applied in more and more institutes, it is mandatory to assess the new technology outcomes and compare them with more traditional approaches before adopting it to wider field, like laparoscopic and open surgeries. Our comprehensive search without limitation to regions, publication types, or languages was done to include the majority of studies related to UBS and minimize publication bias. Besides, we provide the latest information in this area around the world.

## Conclusions

Our systematic review and meta-analysis indicates that use of UBS for UVA during RARP appears to be associated with shorter anastomosis time, operative time and PR time, while it may be equivalent with regard to PL rate, EBL, length of catheterization, or continence rates at 4–6 weeks, 3 months and 6–12 months after surgery. In other word, being a safe suture material, UBS for UVA may be as efficient as CS and have similar functional outcomes for patients with prostate cancer. Nevertheless, even with our rigorous methodology, definitive conclusions are prevented to reach for the inherent limitations of the included studies. Future multicenter, well-designed RCTs with longer follow-up are needed to confirm and update the findings of our research.

## Supporting Information

S1 Table PRISMA checklist. (DOC)

S1 Text A list of full text excluded articles. (DOC)
