# Supplementary material for: The Use of Unidirectional Barbed Suture for Urethrovesical Anastomosis during Robot-Assisted Radical Prostatectomy: A Systematic Review and Meta-Analysis of Efficacy and Safety
Source: PLoS One. 2015 Jul 2;10(7):e0131167. doi: 10.1371/journal.pone.0131167 (PMC4489906; doi:10.1371/journal.pone.0131167)
Supplement: S1 Text — (DOC) [file pone.0131167.s002.doc]

**A List of Full-text Excluded Articles**

**(n=25)**

1. Kilciler M., Tahmaz L., Bedir S., Guler H., Dayanc M. Knotless anastomosis during laparoscopic radical prostatectomy. Journal of Endourology 2009; 23: SUPPL. 1 (A256)

Reasons for exclusion: A conference abstract, not using barbed suture, not proceeding robot-assisted radical prostatectomy(RARP) but laparoscopic radical prostatectomy(LRP).

2. Srivastava A., Sooriakumaran P., Grover S., Rajan S., Eldouaihy Y., Leung R., et al. A novel barbed absorbable suture improves post-prostatectomy reconstruction times. Journal of Endourology. 2010; 24: SUPPL. 1 (A335)
Reasons for exclusion: A meeting abstract, describing the same cohort in one of our included articles.

3. Turpen R.M., Atalah H., Su L. V-Loc™ barbed suture to facilitate vesicourethral anastomosis during robot-assisted laparoscopic radical prostatectomy. Journal of Endourology. 2010; 24: SUPPL. 1 (A334)

Reasons for exclusion: A meeting abstract, not a comparative study.

4. Kaul S., Sammon J., Bhandari A., Peabody J.O., Stricker H., Rogers C., et al. Assistantless urethrovesical anastomosis during Robotic Radical Prostatectomy (RRP) using unidirectional barbed wound closure device. European Urology. Supplements 2010; 9:5 SUPPL. 3 (526)

Reasons for exclusion: A conference abstract, not a comparative study.

5. Trinh Q.D., Sammon J., Kim T.K., Bhandari A., Kaul S., Sukumar S., et al. Urethrovesical anastomosis during robot assisted radical prostatectomy (RARP) using barbed polyglactin suture: Results from a randomized controlled trial. European Urology. Supplements 2011; 10:2 (349)

Reasons for exclusion: A conference abstract, describing the same cohort in one of our included articles.

6. Cisternino A., Latiano C., Rosati A., Gatta F., Croce M., Sebastio N., et al. Robotic radical prostatectomy anastomosis (RRP) assisted by V-Loc™ 180 absorbable closure suture device: Preliminary data and personal surgical experience. Anticancer Research. 2011; 31:5 (1856)

Reasons for exclusion: A conference abstract, patients’ characteristics and data in the control group aren’t available.

7. Pastore A.L., Palleschi G., Autieri D., Ripoli A., Silvestri L., Vacilotto D., et al. Surgical and functional outcomes using a novel device for urethro-vesical anastomosis in extraperitoneal videolaparoscopic prostatectomy: Preliminary data of our experience. Neurourology and Urodynamics. 2011; 30: SUPPL. 1 (9-10)

Reasons for exclusion: A conference abstract, not a comparative research, using barbed suture in extraperitoneal videolaparoscopic prostatectomy but not in RARP.

8. Graversen J.A., Mues A.C., Gupta M., Landman J., Badani K.K. The effect of barbed suture on the posterior reconstruction and urethrovesical anastomosis during robotic assisted laparoscopic prostatectomy. Journal of Endourology. 2011; 25:9 (A4)

Reasons for exclusion: A conference abstract, describing the same cohort in one of our included articles.

9. D'Elia G., Emiliozzi P., Ortolani G., Iannello A., Tuffu G. Knotless three-layer anastomosis during radical robotic prostatectomy. Urology. 2011; 78: 3 SUPPL. 1 (S158)

Reasons for exclusion: A meeting abstract, not a comparative research.

10. Valero R., Schatloff O., Chauhan S., Sivaraman A., Ko Y.H., Palmer K.J., et al. Bidirectional barbed suture: A new technology that could improve results in robotic assisted radical prostatectomy (RARP). European Urology. Supplements 2011; 10:8 (551)

Reasons for exclusion: A meeting abstract, using bidirectional but not unidirectional barbed suture in the study group.

11. Sengupta S., Pan D., Webb D.R. Use of a barbed suture for continuousurethrovesical anastomosis during robot-assisted laparoscopic radical prostatectomy. Asia-Pacific Journal of Clinical Oncology. 2011; 7 SUPPL. 4 (193-194)

Reasons for exclusion: A meeting abstract, not a comparative research, using bidirectional but not unidirectional barbed suture in the study group.

12. Jaramillo F.E., Velásquez J.G., Rios J.F.G., Ossa D.A.V., Cespedes C., Gaviria A. Urethrovesical anastomosis in laparoscopic radical prostatectomy with absorbable barbed suture. Journal of Endourology. 2011; 25 SUPPL. 1 (A324-A325)

Reasons for exclusion: A conference abstract, not a comparative research.

13. Hinata N., Iwamoto H., Inoue S., Matsumoto M., Morizane S., Yao A., et al. Anatomical considerations in nerve sparing robotic radical prostatectomy-tottori experience. Journal of Endourology. 2011; 25 SUPPL. 1 (A310-A311)
Reasons for exclusion: A conference abstract, not a comparative research.

14. Hieda K., Shinmei S., Miyamoto K., Masumoto H., Inoue S., Kobayashi K., et al. Usefulness of V-Loc™180 during laparoscopic radical prostatectomy. Journal of Endourology. 2011; 25 SUPPL. 1 (A90)

Reasons for exclusion: A conference abstract, using barbed suture in LRP but not in RARP.

15. Chin C.M. V-loc suture for posterior reconstruction and vesicourethral anastomosis during robotic prostatectomy. European Urology. Supplements 2012; 11:1 (ev19-ev19a)

Reasons for exclusion: A conference abstract, not a comparative research.

16. Zhuang L., Amling C.Knotless posterior musculofascial plate reconstruction during robotic prostatectomy using V-LocTM sutures. Journal of Urology. 2012; 187:4 SUPPL. 1 (e501)

Reasons for exclusion: A conference abstract, not a comparative research.

17. Zorn K., Trinh Q.-D., Liberman D., ElHakim A. Prospective, randomized use of the vloc vesicourethral anastamosis during robot assisted radical prostatectomy: Long-term followup. Journal of Urology. 2012; 187:4 SUPPL. 1 (e564)

Reasons for exclusion: A conference abstract, describing the same cohort in one of our included articles.

18. Kongchareonsombat W. The perioperative outcomes of knotless wound closure with V-Loc absorbable wound closure device between bladder neck and membranous urethra after removed the prostate gland in laparoscopic radical prostatectomy. preliminary experience in Thailand. Journal of Endourology. 2012; 26 SUPPL. 1 (A311-A312)

Reasons for exclusion: A conference abstract, not a comparative research.

19. Arslan M., Degirmenci T., Gunlusoy B., Gozen A.S., Kozacioʇlu Z., Minareci S. Early results of using new generation polygliconate barbed suture (V-Loc) at the posterior reconstruction and vesicourethral anastomosis in laparoscopic radical prostatectomy. Journal of Endourology. 2012; 26 SUPPL. 1 (A256)

Reasons for exclusion: A conference abstract, using barbed suture in LRP but not in RARP.

20. Tefik T., Khodr M., Karakus S., Salabas E., Ortac M., Tunc M., et al. Barbed suture versus standard monofilament suture for urethrovesical anastomosis during laparoscopic radical prostatectomy. Journal of Endourology. 2012; 26 SUPPL. 1 (A255)

Reasons for exclusion: A conference abstract, using barbed suture in LRP but not in RARP.

21. Ficarra V., Gan M., Borghesi M., Zattoni F., Mottrie A. Posterior muscolofascial reconstruction incorporated into urethrovescical anastomosis during robot-assisted radical prostatectomy. Journal of Endourology. 2012; 26:12 (1542-1545)

Reasons for exclusion: A conference abstract, not a comparative research.

22. Cadeddu J.A. Editorial comment. Journal of Urology. 2011; 185:4 (1280)

Reasons for exclusion: not a research article.

23. Eggener S.E. Editorial comment for Seideman et al. Journal of Endourology. 2011; 25:8 (1247-1248)

Reasons for exclusion: not a research article.

24. Cadeddu JA. Editorial comment. Re: Unfavorable outcomes of laparoscopic pyeloplasty using barbed sutures: a multi-center experience. J Urol. 2014 Aug;192(2):450-1.

Reasons for exclusion: not a research article and not relevant to RARP.

25. [Juan Escudero JU](http://www.ncbi.nlm.nih.gov/pubmed?term=Juan Escudero JU%5BAuthor%5D&cauthor=true&cauthor_uid=23117683), [Ramírez Backhaus M](http://www.ncbi.nlm.nih.gov/pubmed?term=Ramírez Backhaus M%5BAuthor%5D&cauthor=true&cauthor_uid=23117683), [Benedicto Redón A](http://www.ncbi.nlm.nih.gov/pubmed?term=Benedicto Redón A%5BAuthor%5D&cauthor=true&cauthor_uid=23117683), [Ramos de Campos M](http://www.ncbi.nlm.nih.gov/pubmed/?term=Ramos de Campos M%5BAuthor%5D&cauthor=true&cauthor_uid=23117683), [Fabuel Deltoro M](http://www.ncbi.nlm.nih.gov/pubmed/?term=Fabuel Deltoro M%5BAuthor%5D&cauthor=true&cauthor_uid=23117683), [Navalón Verdejo P](http://www.ncbi.nlm.nih.gov/pubmed/?term=Navalón Verdejo P%5BAuthor%5D&cauthor=true&cauthor_uid=23117683), et al. Use of a barbed suture for the urethro-vesical anastomosis during the learning curve of the endoscopic extraperitoneal radical prostatectomy. Arch Esp Urol. 2012; 65(8):752-758.

Reasons for exclusion: comparing the two sutures during endoscopic extraperitoneal radical prostatectomy but not RARP.
